# Widespread Occurrence of Secondary Lipid Biosynthesis Potential in Microbial Lineages

**DOI:** 10.1371/journal.pone.0020146

**Published:** 2011-05-19

**Authors:** Christine N. Shulse, Eric E. Allen

**Affiliations:** 1 Division of Biological Sciences, University of California San Diego, La Jolla, California, United States of America; 2 Scripps Institution of Oceanography, University of California San Diego, La Jolla, California, United States of America; Argonne National Laboratory, United States of America

## Abstract

Bacterial production of long-chain omega-3 polyunsaturated fatty acids (PUFAs), such as eicosapentaenoic acid (EPA, 20:5*n*-3) and docosahexaenoic acid (DHA, 22:6*n*-3), is constrained to a narrow subset of marine γ-proteobacteria. The genes responsible for *de novo* bacterial PUFA biosynthesis, designated *pfaEABCD*, encode large, multi-domain protein complexes akin to type I iterative fatty acid and polyketide synthases, herein referred to as “Pfa synthases”. In addition to the archetypal Pfa synthase gene products from marine bacteria, we have identified homologous type I FAS/PKS gene clusters in diverse microbial lineages spanning 45 genera representing 10 phyla, presumed to be involved in long-chain fatty acid biosynthesis. In total, 20 distinct types of gene clusters were identified. Collectively, we propose the designation of *“secondary lipids”* to describe these biosynthetic pathways and products, a proposition consistent with the “secondary metabolite” vernacular. Phylogenomic analysis reveals a high degree of functional conservation within distinct biosynthetic pathways. Incongruence between secondary lipid synthase functional clades and taxonomic group membership combined with the lack of orthologous gene clusters in closely related strains suggests horizontal gene transfer has contributed to the dissemination of specialized lipid biosynthetic activities across disparate microbial lineages.

## Introduction

Bacteria have evolved the capacity for fatty acid biosynthesis for incorporation into membrane phospholipids in three distinct ways. The most common mechanism is the prototypical type II Fatty Acid Synthase (FAS II), well characterized in *E. coli* [1]. In this system individual enzymatic activities reside on discrete enzyme products, encoded by the **f**atty **a**cid **b**iosynthesis, or *fab*, genes. An alternative pathway, albeit significantly less pervasive in bacterial lineages, is the type I FAS system (FAS I). The canonical pathway found in eukaryotic organisms, FAS I is also found in the Corynebacterineae of the order Actinomycetales [2]. FAS I consists of a large, multifunctional biosynthetic complex containing all enzymatic domains necessary for acyl chain elongation and functional derivatization and is responsible for the production of both membrane phospholipid fatty acyl chains as well as precursor fatty acid molecules for elongation to long-chain mycolic acids in members of the Corynebacteriaceae, Mycobacteriaceae and Nocardiaceae families [3].

A third mechanism of *de novo* fatty acid synthesis coexists with the FAS II in a narrow subset of marine Gammaproteobacteria [4], [5]. This pathway consists of a novel iterative FAS/PKS system and is responsible for the production of long-chain omega-3 polyunsaturated fatty acids (PUFAs) such as eicosapentaenoic acid (EPA, 20:5*n*-3) and docosahexaenoic acid (DHA, 22:6*n*-3) [6], [7], [8]. The genes responsible for bacterial omega-3 PUFA production, designated *pfaA–E*, possess multiple fatty acid biosynthetic enzyme activities as integrated domains within operon-encoded gene products [9].

In addition to polyenoic fatty acyl products, related FAS/PKS gene clusters have been shown to synthesize other specialized long-chain fatty acid products. The C_26_ to C_32_ fatty acid alkyl chains containing hydroxyl or ketone moieties found in the heterocyst glycolipids of filamentous nitrogen-fixing cyanobacteria [10], [11] and the C_22_ to C_26_ fatty acids of phenolic lipids comprising the dormant cysts of the gram-negative bacterium *Azotobacter vinelandii* [12] are both synthesized via an analogous iterative type I FAS/PKS mechanism. Their long chain length, typically containing ≥20 carbons, distinguishes these fatty acyl products from those produced by FAS II (≤18 carbons). We collectively term these specialized lipid products *“secondary lipids”*, to emphasize the accessory nature of these lipid molecules and distinguish these products from those synthesized via core, or primary, fatty acid biosynthetic mechanisms.

All three of these biosynthesis methods make use of highly conserved enzyme activities encoded on integrated domains (FAS I and Pfa synthases) or autonomous monofunctional gene products (FAS II) to accomplish the cycle of condensation, reduction, dehydration, and reduction necessary to produce a final fatty acid product. The biosynthetic reaction sequence of an elongation cycle includes the following activities: 1) Ketoacyl synthase [KS] catalyzes a condensing function responsible for chain-elongation; 2) Ketoacyl reductase [KR] catalyzes the reduction of the carbonyl group to a hydroxyl group; 3) Dehydratase/Isomerase [DH/I] catalyzes the dehydration of the β-hydroxyacyl intermediate generated by KR to a *trans*-2-enoyl derivative and the subsequent isomerization from the *trans*-2 to the *cis*-3 configuration; and lastly 4) Enoyl reductase [ER] catalyzes the reduction of double bond generated by DH to complete the chain elongation process. Other essential activities include acyl carrier protein [ACP] function which tethers the growing fatty acyl chain as a thioester as it is acted upon by other enzyme activities and phosphopantetheinyl transferase [PPTase] activity which converts ACP products from the inactive apo- form to the active holo-form via the posttranslational addition of a 4′-phosphopantetheine prosthetic group from acetyl coenzyme A. Acyltransferases [AT] catalyze the general transfer of a nascent acyl substrate from acyl-CoA to ACP for elongation of the fatty acyl chain. Malonyl-CoA:ACP transacylase [MAT] is a type of acyltranferase that specifically catalyzes the transfer of a 2C malonyl moiety from malonyl-CoA to ACP. A final component, specific to PKSs and Pfa synthases, is the chain length factor [CLF] domain shown to determine the ultimate chain length of the reaction product [13].

Evidence for additional widespread capacity for secondary lipid production potential via the Pfa synthase mechanism in environmental samples has recently been reported [14]. In that study, culture-independent molecular surveys were used to identify 13 novel groups based on KS domain homology from disparate marine habitats. Beyond the marine environment, the phylogenetic extent and ecological breadth of secondary lipid biosynthetic potential has not been investigated.

The current capacity for inexpensive, rapid, whole genome sequencing has allowed for broad genome comparisons among diverse microbial lineages. Here, we expand upon previous studies of the distribution and diversity of secondary lipid production potential in the marine environment [14] by analyzing all sequenced microbial genomes for the presence of FAS/PKS gene clusters homologous to those involved in PUFA secondary lipid biosynthesis. We uncover and classify multiple previously unrecognized FAS/PKS gene clusters in diverse bacterial lineages representing varying physiologies and life histories, significantly expanding the palette and pervasiveness of gene products linked to specialized microbial metabolites.

## Results and Discussion

In the following sections, we describe the diversity and organization of secondary lipid biosynthetic gene clusters identified in this study, first addressing those with characterized products then progressing to novel clusters with uncharacterized products. Additional analyses are presented to support the definition of secondary lipid synthases and differentiate these gene clusters from those involved in PKS or NRPS products based on PPTase domain conservation. Next, we analyze the genomes of secondary lipid synthase containing organisms for the presence of other lipid biosynthetic activities, including FAS II and *ole* gene functions, two systems whose products interact with *pfa* gene products. Lastly, we analyze the ecology and physiological properties of these organisms to provide insight into possible traits unifying secondary lipid production potential and present evidence showing that horizontal gene transfer has aided in the dissemination of these biosynthetic gene clusters.

### Diversity and organization of FAS/PKS gene clusters

**Figure 1:**
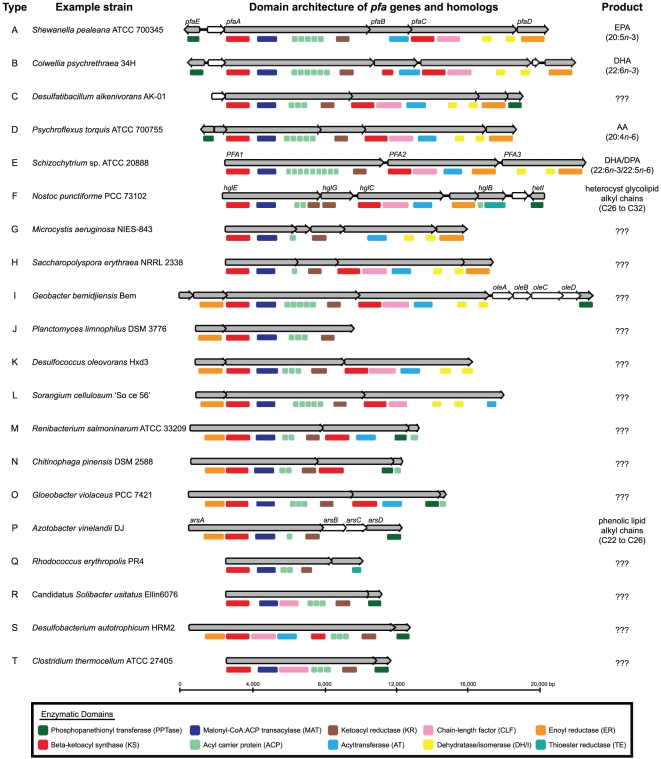
Diversity of *pfa*-like gene clusters. Eighty-six microbial genomes with either the *pfa* gene cluster or a homolog were identified. Type designations are based on KS phylogeny, conserved domain structure and product information, if available.

The presence of multiple acyl carrier protein (ACP) domains in a single gene product is a distinguishing characteristic of Pfa synthases. It has been shown that an increase in the number of ACPs increases the biosynthetic throughput of PUFA product synthesis [15]. Most FAS/PKS gene clusters retrieved in this study contain multiple tandem ACP domains (**Figure 1**). However, although all gene clusters with tandem ACPs are presumed to produce fatty acyl products, not all fatty acyl-producing FAS/PKS gene clusters contain multiple ACPs (e.g. *ars* gene cluster responsible for the production of alkylresorcinols and alkylpyrones in *Azotobacter vinelandii*). Therefore we did not exclude gene clusters containing a single ACP if the domain content and organization was consistent within a candidate FAS/PKS cluster and phylogenetic analysis of the proximal ketoacyl synthase (KS) domain, harbored within the *pfaA* homolog (**Figure 1**), supported a common evolutionary relationship with validated FAS/PKS pathways.

**Figure 2:**
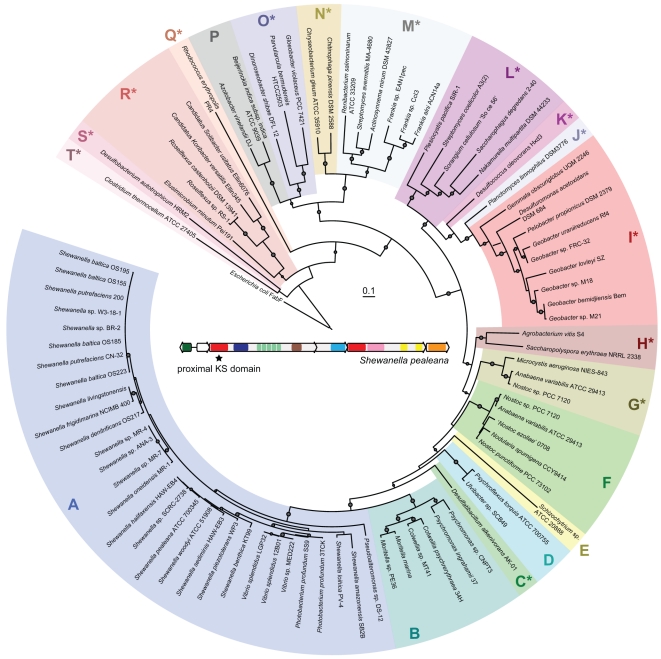
Maximum likelihood phylogenetic tree of proximal keto-acyl synthase protein domains (364 conserved amino acids). Gene cluster Types are colored and given a letter label. Asterisks represent Types first clustered and described in this study. Bootstrap values ≥50% are indicated by dots. The *Escherichia coli* DH10B FabF protein was used as the outgroup.

A total of twenty distinct classes of FAS/PKS gene clusters, designated Types A–T (**Figure 1**), were identified in 86 finished or draft genomes available in GenBank and/or the Joint Genome Institute's Integrated Microbial Genomes (IMG) databases as of February 2011 (2.2% of 3839 genomes analyzed). The division of gene clusters into “Types” was strongly supported by three independent metrics: (i) domain count and organization as analyzed by non-parametric multidimensional scaling (NMDS; **Figure S1**); (ii) phylogenetic analysis of component enzymatic domains in each secondary lipid synthase identified (**Figure 2** and **Figure S2**); and (iii) pathway-product information, if known. Alphabetic ordering of cluster “Types” is based on phylogeny of KS domains (**Figure 2**). All FAS/PKS gene clusters analysed were found to contain at least one KS domain, ACP domain, and ketoacyl reductase (KR) domain, and various combinations of malonyl-CoA:ACP transacylase (MAT), acyltransferase (AT), chain length factor (CLF), dehydratase/isomerase (DH/I), enoyl reductase (ER), and phosphopantetheinyl transferase (PPTase) domains. It is important to investigate both the domain content and organization of each gene cluster as these factors determine the ultimate length and functionality of the chemical product [16]. The NMDS plot (**Figure S1**) is a visual representation of the similarity among all the gene clusters based on these two factors.

#### Secondary lipid synthase types with characterized products

Type A and Type B produce omega-3 PUFAs and are found primarily in γ-proteobacteria of marine origin (**Table 1**). Type A represents the canonical secondary lipid synthesizing gene cluster responsible for eicosapentaenoic acid (EPA, 20:5*n*-3) synthesis and consists of five genes, *pfaABCDE* [9]. Domain order within these genes is highly conserved: *pfaA* [KS-MAT-ACP_(4–6)_-KR], *pfaB* [AT], *pfaC* [KS-CLF-DH_2_], *pfaD* [ER], and *pfaE* [PPTase] (**Figure 1**). An exception to this conservation is found in *Pseudoalteromonas* sp. DS-12 where the PPTase domain is incorporated into *pfaC* [17]. Intriguingly, analysis of the DH domains in *Pseudoalteromonas* sp. DS-12 reveals that the first DH domain is phylogenetically more similar to the second DH domain from all other gene clusters harboring two DH domains, and vice versa (**Figure S2**). This implies that at some point in the evolution of this gene cluster a section of *pfaC* and all of *pfaE* were translocated. It is unknown whether the *Pseudoalteromonas* gene cluster retains the ability to produce EPA [17].

**Table 1:** Membership and description of secondary lipid synthase types with characterized products.

Type	Organism(s)	Unique Characteristics	Product
**A**	*Photobacterium profundum* SS9 and 3TCK | *Pseudoalteromonas* sp. DS-12 | *Shewanella amazonensis* SB2B, *S. baltica* OS155, OS185, OS195 and OS223 *S. benthica* KT99, *S. denitrificans* OS217, *S. frigidimarina* NCIMB 400, *S. halifaxensis* HAW-EB4, *S. livingstonensis*, *S. loihica* PV-4, *S. oneidensis* MR-1, *S. pealeana* ATCC 700345, *S. piezotolerans* WP3, *S. putrefaciens* 200 and CN-32, *S. sediminis* HAW-EB3, *S.* sp. ANA-3, sp. BR-2, sp. MR-4, sp. MR-7, sp. SCRC-2738, sp. W3-18-1, *S. woody*i ATCC 51908 | *Vibrio* sp. MED222, *V. splendidus* 12B01 and LGP32	Marine γ-proteobacteria	Eicosapentaenoic acid (EPA, 20:5*n*-3)
**B**	*Colwellia psychrerythraea* 34H, *C.* sp. MT41 | *Moritella marina*, *Moritella* sp. PE36 | *Psychromonas ingrahamii* 37, *P.* sp. CNPT3	Marine γ-proteobacteria	Docosahexaenoic acid (DHA, 22:6*n*-3)
**D**	*Psychroflexus torquis* ATCC 700755 | *Ulvibacter* sp. SCB49	Marine Bacteroidetes^1^^,^ 2	Arachidonic acid (AA, 20:4*n*-6) and EPA
**E**	*Schizochytrium* sp. ATCC 20888	Osmoheterotrophic Protist	Docosapentaenoic acid (DPA, 22:5*n*-6) and DHA
**F**	*Anabaena variabilis* ATCC 29413 | *Nodularia spumigena* CCY9414 | ‘*Nostoc azollae*’ 0708, *Nostoc punctiforme* PCC 73102, *N.* sp. PCC 7120	Nitrogen fixation; Cyanobacteria	Heterocyst glycolipid alkyl chains (e.g. hexacosanediol, C_26_)
**P**	*Azotobacter vinelandii* DJ | *Beijerinckia indica* subsp. indica ATCC 9039	N_2_ fixation; soil bacteria with varying pH requirements^3^	Phenolic lipid alkyl chains (e.g. behenic acid, C_22_)

1 Bowman JP, McCammon SA, Lewis T, Skerratt JH, Brown JL, et al. (1998) *Psychroflexus torquis* gen. nov., sp. nov., a psychrophilic species from Antarctic sea ice, and reclassification of *Flavobacterium gondwanense* (Dobson et al. 1993) as *Psychroflexus gondwanense* gen. nov., comb. nov. *Microbiol* 144: 1601–1609.

2 Pinhassi J, Zweifel UL, Hagstrom A (1997) Dominant marine bacterioplankton species found among colony-forming bacteria. *Appl Environ Microbiol* 63: 3359–3366.

3 Starkey RL, De PK (1939) A new species of *Azotobacter*. *Soil Sci*ence 47: 329–343.

The Type A cluster was previously found to be conserved in 15 sequenced *Shewanella* strains [18]. In the present study, nine additional *Shewanella* genomes were analyzed and all were found to contain a coherent Type A gene cluster (**Table 1**). The presence of a TypeA *pfa* gene operon in all genomically characterized members of the Shewanellaceae (n = 24) suggests the genetic potential for EPA production is a defining characteristic of this lineage. The *Shewanella* are a genus of Gammaproteobacteria known for their ability to utilize a wide variety of electron acceptors and have been recovered from diverse environmental sources [19], [20], [21]. In addition to the 24 analyzed *Shewanella* genomes, the EPA-synthesizing Type A gene cluster is also found in three *Vibrio* genomes (*Vibrio* sp. MED222, *V. splendidus* 12B01, and *V. splendidus* LGP32), two *Photobacterium* genomes [9] and one *Pseudoalteromonas* genome [17].

Type B represents the docosahexaenoic acid (DHA)-producing *pfa* gene cluster, which differs from the EPA-producing *pfa* cluster by the insertion of an additional KS domain in *pfaB* (**Figure 1**). An active site cysteine was found to be absent in all Type B *pfaB* KS domains examined in the current study. A recent study suggested that this *pfaB* KS domain may play a role in determining the final PUFA end product, as *E. coli* transformed with *pfaA*, *pfaC*, *pfaD* and *pfaE* from an EPA-producer and *pfaB* from a DHA-producer made both EPA and DHA [22]. The Type B gene cluster is found exclusively in marine γ-proteobacteria representing three genera: *Colwellia* [23], *Psychromonas* [24], and *Moritella* [8]. Although the Type B gene cluster retains a completely conserved domain structure in the genomes of the *Colwellia*, *Moritella*, and *Psychromonas* and the proximal *pfaA* KSs from these species group together at 74% amino acid sequence identity, *Colwellia* and *Moritella* are capable of producing significant quantities of DHA while the *Psychromonas* that have been investigated either do not produce DHA or produce it only in trace quantities [25], [26].

Type D is found in two Bacteriodetes strains (**Table 1**), of which *Psychroflexus torquis* ATCC 700755 is known to produce arachidonic acid (AA, 20:4*n*-6) and EPA [27]. The Type D gene cluster has the same domain content as the Type A EPA-producing gene cluster, however the AT domain is rearranged and domains reside on split or fused gene products – the *pfaA* homolog is split into two genes, designated *pfaA1* [KS-AT-ACP_5_] and *pfaA2* [KR], and *pfaB* and *pfaC* are fused into one gene, *pfaBC* [KS-CLF-AT-DH_2_] (**Figure 1**).

The Type E gene cluster is found in the marine thraustochytrid *Schizochytrium* sp. ATCC 20888 (order Labyrinthulida) and is involved in the production of DHA and the omega-6 PUFA docosapentaenoic acid (DPA, 22:5*n*-6) [6]. This gene cluster consists of three genes, designated *PFA1*, *2*, and *3*, and contains the same domains found in the five genes of the Type A cluster. Although the Type E gene cluster is clearly homologous to the Type A and B clusters, differences do exist. For example, in contrast to the Type A and B gene clusters, each containing one ER domain, the Type E cluster contains two, one located on *PFA2* and the other on *PFA3* (**Figure 1**). Sequence composition analyses indicate that these *Schizochytrium* ER domains share 89% identity at the amino acid level. Thus one of the ER domains may have resulted from a duplication and translocation event after transfer of an ancestral Pfa synthase gene cluster into the *Schizochytrium* genome. In addition to the Type E cluster for PUFA synthesis, *Schizochytrium* sp. ATCC 20888 also contains the canonical eukaryotic PUFA biosynthesis pathway, involving elongase and desaturase enzymes [28]. The presence of a bacterial Pfa synthase in *Schizochytrium* is significant as it suggests evidence of lateral gene transfer between a bacterium and eukaryote. The integration and retention of a Pfa synthase in the *Schizochytrium* genome may contribute to the high PUFA content observed in certain labyrinthulids [29], [30]. DHA- and DPA-producing thraustochytrids have been isolated from marine environments around the globe [31], although the relative contribution of each pathway to PUFA synthesis has not been determined in these strains.

Type F is found in the genomes of five nitrogen-fixing cyanobacteria. First characterized in the cyanobacterium *Nostoc punctiforme* strain ATCC 29133, the *hgl* genes, for *h*eterocyst *gl*ycolipid [10], were shown to be involved in the production of the lipid moeity of heterocyst glycolipids. The other four cyanobacteria containing the Type F gene cluster, *Nostoc azollae* 0708, *Anabaena variabilis* ATCC 29413, *Nodularia spumigena* CCY 9414, and *Nostoc* sp. PCC 7120, also form heterocysts for nitrogen fixation. The Type F gene cluster is remarkably similar to Types A and B. One notable difference is the fusion of some of the domains contained on *pfaB* and *pfaC* on a single gene, *hglC* [KS-CLF-AT]. Additionally, the Type F cluster lacks the DH/I domains typically found on *pfaC*.

The final Type with a characterized pathway for which the chemical product has been verified is the Type P gene cluster, found in the genomes of two nitrogen-fixing Proteobacteria. This synthase is responsible for the production of the alkyl moiety of phenolic lipids in the cyst-forming Gammaproteobacterium *Azotobacter vinelandii* [12]. The Type P gene cluster is also found in the Alphaproteobacterium *Beijerinckia indica* subsp. Indica ATCC 9039, which has not been found to produce cysts or phenolic lipids [32].

#### Secondary lipid synthase types with uncharacterized products

In addition to the above pathways with characterized products, 14 additional gene clusters homologous to the *pfa* genes were discovered with putative fatty acyl end products (**Table 2**). The Type C cluster has been found in only one sequenced bacterial genome, that of the Deltaproteobacterium *Desulfatibacillum alkenivorans* AK-01, which has not been reported to produce PUFAs. This gene cluster contains a *pfaBC* fusion [KS-CLF-AT-DH_2_] and *pfaD* [ER] is located at the 3′ end of the gene cluster.

**Table 2:** Membership and description of secondary lipid synthase types with uncharacterized products.

Type	Organism(s)	Unique Characteristics
**C**	*Desulfatibacillum alkenivorans* AK-01	Isolated from sediments^1^
**G**	*Anabaena variabilis* ATCC 29413 | *Microcystis aeruginosa* NIES-843 | *Nostoc* sp. PCC 7120	Cyanobacteria
**H**	*Agrobacterium vitis* S4 | *Saccharopolyspora erythraea* NRRL 2338	Plant pathogen^2^; Soil bacterium^3^
**I**	*Desulfuromonas acetoxidans* DSM 684 | *Gemmata obscuriglobus* UQM 2246 | *Geobacter bemidjiensis* Bem, *G. lovleyi* SZ, *G.* sp. FRC-32, sp. M18, sp. M21, *G. uraniireducens* Rf4 | *Pelobacter propionicus* DSM 2379	Deltaproteobacteria, except *G. obscuriglobus* (Planctomycete)
**J**	*Planctomyces limnophilus* DSM 3776	Planctomycete
**K**	*Desulfococcus oleovorans* Hxd3	Sulfate-reducer^4^
**L**	*Nakamurella multipartita* DSM 44233 | *Plesiocystis pacifica* SIR-1 | *Saccharophagus degredans* 2-40 | *Sorangium cellulosum* ‘So ce 56’ | *Streptomyces coelicolor* A3(2), *S. ghanensis* ATCC 14672	Actinobacteria, Deltaproteobacteria, Gammaproteobacteria
**M**	*Actinosynnema mirum* DSM 43827 | *Frankia alni* ACN14a, *F.* sp. CcI3, *F.* sp. EAN1pec | *Renibacterium salmoninarum* ATCC 33209 | *Streptomyces avermitilis* MA-4680	*Frankia* = nitrogen fixers
**N**	*Chitinophaga pinensis* DSM 2588 | *Chryseobacterium gleum* ATCC 35910	Bacteroidetes
**O**	*Dinoroseobacter shibae* DFL 12 | *Gloeobacter violaceus* PCC 7421 | *Parvularcula bermudensis* HTCC2503	*Gloeobacter violaceus* PCC 7421 produces PUFAs (18∶2, 18∶3); desaturases^5^
**Q**	*Rhodococcus erythropolis* PR4	Alkane-degrader^6^
**R**	Candidatus *Koribacter versatilis* Ellin345 | Candidatus *Solibacter usitatus* Ellin6076 | *Elusimicrobium minutum* Pei191 | *Roseiflexus castenholzii* DSM 13941, *R.* sp. RS-1	
**S**	*Desulfobacterium autotrophicum* HRM2	Sulfate-reducer^7^
**T**	*Clostridium thermocellum* ATCC 27405	Thermophilic, anaerobic^8^

1 So CM, Young LY (1999) Isolation and characterization of a sulfate-reducing bacterium that anaerobically degrades alkanes. Applied and Environmental Microbiology 65: 2969–2976.

2 Ophel K, Kerr A (1990) *Agrobacterium vitis* sp. nov. for Strains of *Agrobacterium* biovar 3 from Grapevines. International Journal of Systematic Bacteriology 40: 236–241.

3 Labeda DP (1987) Transfer of the Type Strain of *Streptomyces erythraeus* (Waksman 1923) Waksman and Henrici 1948 to the Genus *Saccharopolyspora* Lacy and Goodfellow 1975 as *Saccharopolyspora erythraea* sp. nov., and Designation of a Neotype Strain for *Streptomyces erythraeus*. International Journal of Systematic Bacteriology 37: 19–22.

4 Aeckersberg F, Bak F, Widdel F (1991) Anaerobic oxidation of saturated hydrocarbons to CO2 by a new type of sulfate-reducing bacterium. Archives of Microbiology 156: 5–14.

5 Chi XY, Yang QL, Zhao FQ, Qin S, Yang Y, et al. (2008) Comparative Analysis of Fatty Acid Desaturases in Cyanobacterial Genomes. Comparative and Functional Genomics.

6 KomukaiNakamura S, Sugiura K, YamauchiInomata Y, Toki H, Venkateswaran K, et al. (1996) Construction of bacterial consortia that degrade Arabian light crude oil. Journal of Fermentation and Bioengineering 82: 570–574.

7 Brysch K, Schneider C, Fuchs G, Widdel F (1987) Lithoautotrophic growth of sulfate-reducing bacteria, and description of *Desulfobacterium autotrophicum* gen. nov., sp. nov. Archives of Microbiology 148: 264–274.

8 McBee R (1954) The characteristics of *Clostridium thermocellum*. Journal of Bacteriology 67: 505–506.

The Type G gene cluster, like the characterized Type F cluster, is found in cyanobacterial genomes. In fact, in the case of *Anabaena variabilis* ATCC 29413 and *Nostoc* sp. PCC 7120, the Type F and Type G gene clusters coexist in the same genome. Notably, the Type G gene cluster is always found to have two uncharacterized type I PKS genes directly upstream. The product of the Type G gene cluster may interact with the product of the linked type I PKS genes, producing a hybrid polyketide/fatty acid product. A precedent for the interaction of products from a PKS and a FAS/PKS exists, for example, in the case of phenolic lipid production in *Azotobacter vinelandii* (Type P). In this case, the phenolic functional group is produced by two type III PKS genes, which are flanked on both sides by the Type P FAS/PKS genes responsible for the production of the C_22–26_ fatty acid moiety of phenolic lipids. A similar head/tail functional group production relationship could be occurring between the Type G gene cluster and the upstream PKS genes.

*Agrobacterium vitis* S4 (class/subphylum Alphaproteobacteria) and *Saccharopolyspora erythraea* NRRL 2338 (phylum Actinobacteria) harbor the Type H gene cluster (**Table 2**). This gene cluster is defined by a split *pfaA* and a fused *pfaBC* homolog (**Figure 1**).

Type I contains a *pfaBC* fusion and is found in multiple genera of Deltaproteobacteria and one Planctomycete (**Table 2**). The Type J gene cluster is only found in one genome, that of *Planctomyces limnophilus*. It consists of just two genes, a *pfaD* homolog [ER] followed by a *pfaA* homolog [KS-MAT-ACP_3_-KR] (**Figure 1**). Phylogenetically, the Type J KS domain is closely related to the KS domain from the Type I gene cluster (**Figure 2**). This apparently partial gene cluster could have arisen after a partial transfer of a more complete Type I-like cluster. The Type K gene cluster also has a similar configuration to the Type I cluster (**Figure 1**) and is found in a single Deltaproteobacterium, *Desulfococcus oleovorans* Hxd3 (**Table 2**).

The Type L gene cluster is distinguished by a fused *pfaBC* [KS-CLF-DH_2_-AT] homolog, and a *pfaD* [ER] homolog at the 5′ end of the gene cluster. It has been suggested that this gene cluster plays a role in the production of polyunsaturated fatty acids in *Streptomyces coelicolor* A3(2) [33] however PUFA production has not been demonstrated in this strain.

The Type M cluster is conserved in all three finished genomes from strains representing the nitrogen-fixing genus *Frankia* (phylum Actinobacteria) (**Table 2**). This is especially significant given that these genomes range in size from 5.43 to 9.04 Mbp, indicating extensive genome expansion, contraction, and adaptation to specific niches [34].

The Type N cluster is found in the chitin-degrading species *Chitinophaga pinensis* DSM 2588, which forms spherical “resting bodies,” or microcysts, upon aging [35], as well as the clinical isolate *Chryseobacterium gleum* ATCC 35910. The composition of the *C. pinensis* DSM 2588 “resting body” has not been determined, and it is possible that the product of the Type N plays a structural role in these differentiated cells, a proposal consistent with the structural role of phenolic lipids (Type P) in differentiated cysts of *A. vinelandii* [36]. However *Chryseobacterium gleum* ATCC 35910 is not known to produce differentiated cell types. The Type O gene cluster is found in the genomes of two marine Alphaproteobacteria, *Dinoroseobacter shibae* DFL 12 and *Parvularcula bermudensis* HTCC2503, and the rock-dwelling cyanobacterium *Gloeobacter violaceus* PCC 7421. The Type Q gene cluster is found in a single bacterial strain, *Rhodococcus erythropolis* PR4 (phylum Actinobacteria) (**Table 2**).

The Type R cluster is dispersed throughout several diverse microbial phyla, including the Acidobacteria (Candidatus *Koribacter versatilis* Ellin345 and Candidatus *Solibacter usitatus* Ellin6076), Elusimicrobia (*Elusimicrobium minutum* Pei191), and Chloroflexi (*Roseiflexus castenholzii* DSM 13941 and *Roseiflexus* sp. RS-1). The Type R cluster consists of one *pfaA* homolog containing the domain architecture KS-MAT-CLF-ACP_(3–6)_-KR followed by a downstream gene encoding phosphopantetheinyl tranferase (PPTase) activity. Although there is strong evidence that this gene cluster shares an evolutionary origin (**Figure 2**), it is possible that the fatty acyl products are modified to produce different end products in these physiologically diverse and ecologically distinct species. The Type R core gene cluster homologous to the *pfa* genes is often associated with other genes involved in lipid production. For example, in *Korebacter versatilis* Ellin345, the *pfaA* and *pfaE* homologs are separated by a gene encoding part of the acetyl-CoA carboxylase (ACC) complex, which catalyzes the carboxylation of acetyl-CoA to produce malonyl-CoA for fatty acid biosynthesis. This intervening gene is absent in the remaining four gene clusters comprising Type R.

Types S and T are each found in single representatives (**Table 2**).

### Analysis of phosphopantetheinyl transferase domains

One characteristic distinguishing PKSs from lipid synthases is the molecular sequence of the associated PPTase products responsible for posttranslational modification and activation of ACP domains [37]. To provide additional evidence that we have identified novel secondary lipid synthases rather than PKSs, we investigated PPTase domain diversity. The informative variable motifs are known as P0, P1a, and P1b in PUFA-specific PPTases and 1A, P1a′, and P1b′ in PKS/NRPS-specific PPTases. Secondary lipid synthase Types A, B, C, D, F, I, L, M, N, O, P, R, S, and T most often contain an associated PPTases in direct proximity to the Pfa synthase. Of these, Types A, B, C, D, F, I, and L contain the PUFA-associated P0 motif – defined as (L/V)Rx(L/V)LS in [37] and modified to hRxhLS in this comprehensive study of 69 secondary lipid-associated PPTases, where h = hydrophobic residue (**Figure S3**). Importantly, the PPTase from the Type P gene cluster, known to be involved in the production of phenolic lipids, does not contain the P0 motif. Consequently, the P0 domain may not be necessary for the production of secondary lipids in general. All secondary lipid Types investigated contain the PUFA-associated P1a motif – defined as K(G/D)KP in [37] and modified to x(G/D)xP in this study – rather than the PKS-related P1a′ motif. Exceptions were the *Photobacterium* PPTases which were previously shown to be EntD-like PPTases [37]. EntD in *E. coli* is involved in the production of siderophore enterobactin [38] and is included in the PKS/NRPS-producing PPTase group. Examination of the final domain region revealed that Types A, B, C, D, F, I, and L contain the P1b domain – FNxSH in [37] and modified to (F/S)NxSH in this study – while Types M, N, O, P, R, T, and S contain the P1b′ domain – GSIxH in [37] and modified to hShxH in this study (**Figure S3**). This bifurcation between Types A–L and Types M–T is also reflected in the KS tree (**Figure 2**) and could have implications for the type of end product produced from these gene clusters.

### Relationship of secondary lipid pathways with FAS II

The relationship between secondary lipid pathways and FAS II is of interest as both pathways draw from the same intracellular pool of precursor molecules for their biosynthetic activities. All bacteria possessing the archetypal Pfa synthase (Type A and B) also possess a complete FAS II and generally produce polyunsaturated fatty acids as a small percentage of their total fatty acids, with saturated and monounsaturated fatty acids comprising the majority of their phospholipids [25]. However, several interesting exceptions exist among the bacteria harboring other putative secondary lipid synthases. The genome of the fish pathogen *Renibacterium salmoninarum* (Type M) lacks both *fabA* [DH/I] and *fabZ* [DH] homologs, at least one of which is necessary to perform the dehydration step involved in the biosynthesis of both saturated and monounsaturated fatty acids [39]. Based on genome analysis and growth capabilities it has been suggested that this strain requires exogenous fatty acids, possibly scavenged from its host, for incorporation into membrane phospholipids [40]. Consequently, the Type M pathway present in *R. salmoninarum* genome likely represents the only complete fatty acid biosynthesis pathway present in this strain. Likewise, *Desulfobacterium autotrophicum* HRM2 (Type S) is also missing *fabA* and *fabZ* homologs. Unlike the pathogenic *R. salmoninarum*, the completely oxidizing sulfate-reducing bacterium *D. autotrophicum* is free-living. Presumably, it could incorporate the fatty acids typically used as carbon sources into its membrane phospholipids. Given that *D. autotrophicum* harbors an incomplete FAS II, it is possible that the Type S secondary lipid synthase gene cluster is functioning as the “core” fatty acid synthase for this organism, thus the main producer of fatty acids for incorporation into phospholipids.

### Co-occurrence of secondary lipid synthesis pathways with olefin-producing genes

Several major outstanding questions exist regarding the products synthesized by uncharacterized secondary lipid synthases described here (Types C, G, H, I J, K, L, M N, O, Q, R, S, T). It has previously been demonstrated that *pfa* gene products are necessary for the production of olefinic hydrocarbons in *Shewanella onedensis* MR-1 [41]. *pfa* genes and several secondary lipid synthase types described here co-occur with the *ole* genes responsible for the head-to-head condensation of fatty acyl products and the formation of olefinic neutral lipids [42], in 44 of the 80 (55%) sequenced genomes examined (*Pseudoalteromonas* sp. DS-12, *Shewanella livingstonensis*, *Shewanella* sp. BR-2, *Shewanella* sp. SCRC-2738, *Moritella marina*, and *Schizochytrium* sp. ATCC 20888 were excluded from this analysis as their genomes have not been sequenced). Furthermore, *Geobacter bemidjiensis* produces the same olefinic hydrocarbon, hentriacontanonaene (C_31_H_46_), found in Shewanella strains harboring the *pfa* gene cluster. Therefore, we suggest that the Type I “*pfa*-like” secondary lipid synthase gene cluster in the Deltaprotoebacteria and Planctomycete strains may provide precursors necessary for olefin biosynthesis. Additionally, it is possible that the Type L cluster contributes to the production of olefinic hydrocarbons. *Nakamurella multipartita* DSM 44233 and *Plesiocystis pacifica* SIR-1 both contain *ole* genes [42].

These *ole* genes are often linked with the FAS/PKS gene clusters but can also reside elsewhere in the genome. In cases where unlinked *pfa* and *ole* genes exist, the transfer of these genes into the genomes was not coordinated, as bacteria possessing the same “Type” of *pfa* gene cluster often have differing *ole* gene configurations. An interesting question then arises regarding the primacy of these two biosynthetic gene clusters. One possibility is that the *pfa* homologs were originally producing a different product and were co-opted for the production of precursors for olefin biosynthesis over evolutionary time. It is also possible that the *pfa* homologs retain the ability to produce a stand-alone product and only contribute to olefin production under certain environmental conditions. Very little is known regarding the regulation and synthesis of olefin products.

### Ecology of microorganisms possessing secondary lipid production potential

The microorganisms harboring the *pfa* genes and homologs belong to diverse phyla throughout the bacterial domain, exhibiting varied ecophysiologies and lifestyle strategies. Examination of this metadata provided important insight into the ecological basis of secondary lipid synthesis across the spectrum of biosynthetic diversity examined. A permutational analysis of variance (PERMOVA) was used to rigorously test the association between gene cluster Type and life history traits, for which there was adequate replication (n≥5, Types A, B, F, I, L, M, and R) (**Figure S4**). All gene clusters differed from each other in terms of life history traits (PERMANOVA pair-wise tests, P = ≤0.004), with the exception that L did not differ from M or R, and M did not differ from R (P = ≥0.05). Overall allocation success across all seven gene clusters equaled 60.6%, with all genes having a more distinct set of life history traits than expected by chance alone, with the exception of R. Type I was associated with being an obligate anaerobe. Many of the known fatty acid products produced by characterized Types are known to play a role in excluding oxygen from the cell (e.g. heterocyst glycolipids), or have been shown to provide antioxidative functions to the cell [43]. Consistent with these functions, the product of the Type I *pfa* homologs in the Deltaproteobacteria may play a role in protecting these sensitive cells from lethal levels of oxygen in the environment. A complete PERMOVA analysis on all secondary lipid synthase types is presented in **Figure S5**.

### Contribution of horizontal gene transfer to the dissemination of secondary lipid synthase pathways

**Figure 3:**
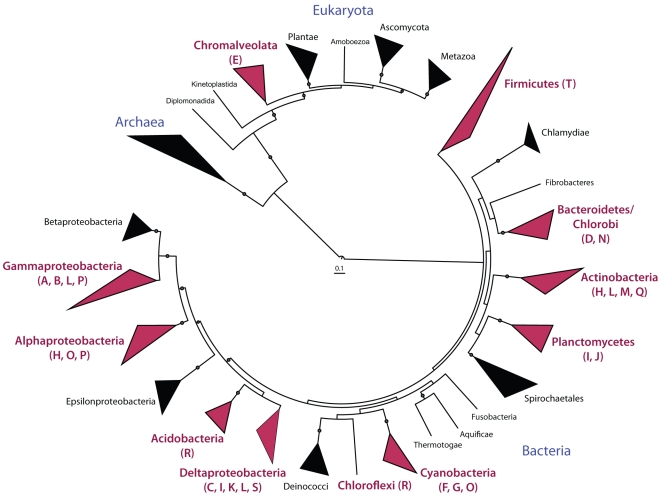
Phylogenetic distribution of *pfa* gene clusters and homologues. Clades in red contain putative secondary lipid gene clusters. Tree modified from the Interactive Tree of Life.

**Figure 4:**
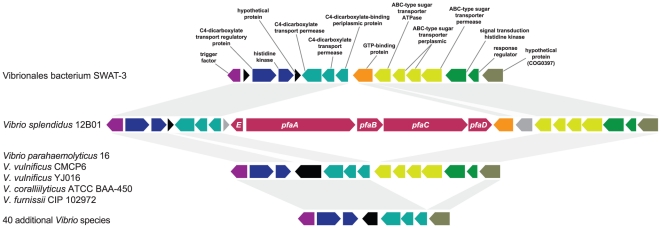
Plot of *Vibrio splendidus* 12B01 *pfa* region versus corresponding genomic regions of multiple *Vibrio* species. Gray tracts indicate regions of homology between genomes. The *pfa* genes are labeled in the *Vibrio splendidus* 12B01 genome and are absent in the other *Vibrio* genomes.

Horizontal gene transfer (HGT) may help explain the patchy distribution of secondary lipid pathways among bacterial and eukaryotic phyla. Specifically, multiple secondary lipid Types may be found in the same phyla, while a single Type may be found across several phyla (**Figure 3**). To examine the possibility of HGT, all species of the same genus having an identified secondary lipid synthase gene cluster with two or more sequenced genomes were analyzed for evidence of HGT. For example, the *pfa* gene cluster (Type A) is present in three strains of *Vibrio* but absent in many closely related *Vibrio* genomes. A comparison of the *pfa* gene region of *Vibrio splendidus* 12B01 and corresponding regions of the genomes of multiple *Vibrio* species is shown in **Figure 4**. The two genomic regions immediately flanking the *pfa* gene cluster show 93% and 87% identity at the nucleotide level, respectively, to their homologs in the Vibrionales bacterium SWAT-3 genome. The *pfa* gene cluster clearly shows a perfect insertion characteristic of a genomic island.

The Type I gene cluster has an especially sporadic distribution among the Deltaproteobacteria. Of the two sequenced *Pelobacter* strains, *Pelobacter propionicus* DSM 2379 harbors a Type I cluster while *Pelobacter carbinolicus* DSM 2380 does not. Likewise, of the nine sequenced *Geobacter*, only six contain the Type I secondary lipid synthase. Furthermore, omega-3 PUFA production in marine Labyrinthulid protists (Eukaryota) of the genus *Schizochytrium* is known to proceed via the same FAS/PKS-related pathway providing additional evidence implicating HGT as an important contributor to chemical diversification [6], [28].

It has been noted that the *pfaA* homolog in *Elusimicrobium minutum* Pei191 (Type R) has an aberrant GC content (46%) compared to the rest of the genome (39%), prompting the suggestion that it entered the genome through horizontal gene transfer [44]. To further investigate the potential novelty of these gene clusters within their host genomes, DNA compositional analyses were performed (%GC analysis and AlienHunter [45]). No conclusive evidence based on DNA compositional metrics was revealed, indicating these gene clusters are not aberrant within their respective genomes. Our analyses of all secondary lipid synthase gene clusters indicate that the GC content of the *pfaA* homolog is consistently higher than the genome average (data not shown), most likely a reflection of the amino acid composition of these proteins. While these results do not rule out possible ancient acquisition of these pathways, comparative genomic analysis, as presented above, provides better resolution in support of horizontal acquisition of these gene clusters. Improved genomic sampling within individual phylogenetic groups will enhance our ability to resolve the evolutionary history and mobility of secondary lipid production potential.

### Conclusions

Secondary lipid synthase gene clusters have been detected in 10 microbial phyla, representing 86 species across two domains of life. The genetic potential to produce long-chain fatty acids via a FAS/PKS mechanism appears to be scattered throughout the bacterial domain and has been co-opted by some Eukarya. Results presented here demonstrate that these biosynthetic pathways are not relegated solely to a narrow group of marine bacteria, as previously believed. Instead, this third mechanism of bacterial fatty acid synthesis may be involved in the production of specialized lipid products across the bacterial tree. As additional genomes are sequenced and their physiologies explored, additional bacterial lineages harboring novel secondary lipid pathways and producing novel fatty acyl products will be revealed. The linking of these pathways to chemical products and determining the physiological role of these biosynthetic processes is a crucial next step.

## Methods

### Identification of FAS/PKS candidate sequences in sequenced microbial genomes

Genomes were downloaded from NCBI and the Joint Genome Institute's Integrated Microbial Genomes (IMG) database (http://img.jgi.doe.gov). A local TBLASTN of *pfa* genes known to produce EPA and DHA was performed against the downloaded genomes and results were curated by e-value (≤1e-30) to identify possible candidate sequences. To minimize false positive hits (e.g. polyketide synthase or nonribosomal peptide synthase genes), a refined set of query sequences was designed to exploit the conserved domain architecture of secondary lipid synthase gene products (e.g. PfaA–E). A subsequent TBLASTN of individual domains (KS, MAT, ACP, KR, DH, ER) was performed. These analyses provided a broad list of candidate genes. The most informative query sequences were tandem ACP sequences, a hallmark characteristic of secondary lipid products. All candidate genomes and specific gene neighborhoods were inspect for adjacent genes using the sequence visualization tool Artemis [46] and via IMG's Gene Ortholog Browser tool. Protein sequences were annotated using InterProScan [47] and manually curated. **File S1** is presented as an Excel worksheet and provides a complete list of annotations and GenBank accession numbers for each protein sequence investigated in this study.

### Phylogenetic analyses

Proximal KS amino acid sequences were aligned using MAFFT [48] and viewed and edited in Jalview [49]. Poorly aligned and/or divergent positions were excluded using Gblocks [50] with a minimum block of five and allowed gap positions equal to half. The best model for the resulting 364 amino acid alignment was evaluated using ProtTest [51], a program for selecting a model of protein evolution that uses PHYML [52] and PAL [53]. ProtTest chose LG+G+F based on Akaike criterion. A maximum likelihood phylogenetic tree was constructed using RAxML rapid bootstrapping [54] through the CIPRES portal [55] cluster at the San Diego Supercomputer Center. The phylogenetic tree graphic was produced using the Interactive Tree of Life [56]. The DH tree was constructed in an identical manner from the full alignment however using the LG+G model.

### Multidimensional scaling analysis

Component domains from each gene cluster were letter coded (e.g. KS-MAT-ACP_2_-KR-ER-PPTase becomes ABCCDEF). Coded gene clusters were aligned using MAFFT and viewed and edited in Jalview. The alignment was used to generate a pairwise distance matrix in MOTHUR [57] and subsequently converted to a similarity matrix. This similarity matrix was imported into Primer 6 [58] and used to create a multidimensional scaling (MDS) plot, using default parameters (25 random starts, Krustal fit scheme of 1, and a minimum stress value of 0.01). Subsequently, a cluster analysis of the data was performed, using average group linkage, and overlaid in order to define “Types” based on domain content and organization.

### Permutational analysis of variance and canonical analysis of principal coordinates

A set of 33 life history traits were allocated to each bacterial species to create a binary matrix. A permutational analysis of variance (PERMANOVA) [59], [60] was used to test the association between gene cluster type and bacterial life history traits. The PERMANOVA was based on unrestricted permutations of the raw data and a partial sums of squares. To visualize and test which life history traits were associated with which gene clusters we used a constrained canonical analysis of principal coordinates (CAP) [61], [62]. CAP analyses were based on 10,000 random permutations of the raw data and a Bray-Curtis similarity matrix. Individual life history traits that might be responsible for any differences in multivariate space were investigated by calculating Spearman Rank correlations of canonical ordination axes with the original genera variables. Traits with strong correlations (defined as ≥0.4 in this study) were then overlaid as a bi-plot. We used the leave-one-out procedure in the CAP analysis to calculate allocation success for each *a priori* defined gene cluster. This essentially gave us a measure of distinctness for the life history traits associated with each gene cluster. Allocation success was considered indicative of a more distinct set of life history traits than expected by chance alone when values exceeded 14.3%. This threshold came from the possibility of each individual observation having a 14.3% chance of being placed into one of the 7 *a priori* defined groups (cluster Types). As replication within each *a priori* defined gene cluster group varied (ranging from n = 1 to n = 30), formal tests were only run on those gene clusters for which n≥5 (A, B, F, I, L, M, R).

## Supporting Information

Figure S1 Multidimensional scaling (MDS) plot of similarity matrix of domain order and count in *pfa* gene clusters and homologues. See File S1 for numerical key describing species abbreviations.
(EPS)

Figure S2 Maximum likelihood phylogenetic tree of dehydratase/isomerase protein domains (158 amino acid alignment). Gene cluster Types are colored and given a letter label. Asterisks represent Types first clustered and described in this study. Bootstrap values ≥50% are indicated by dots. The *E. coli* DH10B FabA protein was used as the outgroup.
(EPS)

Figure S3 Multiple sequence alignment showing sections of PPTase domains from representative organisms. Conserved domains are labeled at the top of the alignment. Motifs P2 and P3 do not show a clear pattern of variation among different secondary lipid types.
(EPS)

Figure S4 CAP ordination of the similarity among seven gene clusters (A, B, F, I, L, M, R) based on 33 life history traits. Group centroids are displayed for each gene cluster to ease interpretation. Vector lines in the bi-plot represent Spearman Rank correlations, with the direction indicating the relationship of each trait to the gene clusters in multivariate space. The length of each vector line is proportional to the strength of the correlation, with the blue circle showing the threshold for a correlation of one.
(EPS)

Figure S5 CAP ordination of the similarity among all gene clusters (A–T) based on 33 life history traits. Group centroids are displayed for each gene cluster to ease interpretation. Vector lines in the bi-plot represent Spearman Rank correlations, with the direction indicating the relationship of each trait to the gene clusters in multivariate space. The length of each vector line is proportional to the strength of the correlation, with the blue circle showing the threshold for a correlation of one.
(EPS)

File S1 Excel spreadsheet providing GenBank accession numbers and domain annotations for putative secondary lipid synthase genes. Numbers in column B provide a key for numerical abbreviations used in Figure S1.
(XLS)
